# Patient Opinions and Side Effects Before and After General Anesthesia for Surgery

**DOI:** 10.7759/cureus.53755

**Published:** 2024-02-07

**Authors:** Mohammad Usman, Austin Huang, Laurence Stolzenberg, Martin Clemmons, Justin G Hovey, Gordon MacGregor

**Affiliations:** 1 Psychiatry, Alabama College of Osteopathic Medicine, Dothan, USA; 2 Internal Medicine, Alabama College of Osteopathic Medicine, Dothan, USA; 3 Radiology, Alabama College of Osteopathic Medicine, Dothan, USA; 4 Pediatrics, Alabama College of Osteopathic Medicine, Dothan, USA; 5 Pharmacology, Orlando College of Osteopathic Medicine, Orlando, USA

**Keywords:** propofol, sedation, surgery, general anesthesia, anesthesia

## Abstract

As the number of surgical procedures performed the world over continues to increase, so does the number of anesthetic procedures needed for those surgeries to occur. While much thought and research has been focused on the perspective of the anesthesiologist, little has been explored from the perspective of the patient receiving anesthesia. The purpose of this study was to explore the general public’s opinions and experiences of general anesthesia, as well as any change in their perception after having undergone a procedure requiring it. We decided that further inquiry into the subject was warranted, and we decided to run an online Qualtrics survey in order to make that inquiry.

The key takeaway from our online anonymous survey shows that there is a significant amount of anxiety related to anesthesia, but that most people specify a large decrease in said anxiety after having undergone the procedure. Noticeably, people were made more comfortable by discussing anesthesia with people who had lived through the experience, and people believed they would be significantly comforted by the presence of therapy animals prior to beginning their procedures.

We hope that our exploratory research will promote future research into this topic in order to improve the healthcare outcomes of a significant number of patients. We believe that this data has opened up many potential avenues for further exploration and research, as well as potentially being able to guide surgical practice.

## Introduction

General anesthesia (GA) is a specific form of anesthesia with the goal of inducing unconsciousness in a patient undergoing a major surgical procedure [1]. It has been estimated that about 234.2 million major surgical procedures occur every year worldwide, with 40-50 million in the USA alone [2,3]. We believe it is important to gain insight into the patient's perspective before and after undergoing GA. We specifically want to investigate common sources of fear and anxiety and what could have been done to make patients feel more comfortable. The collection of this data will aid in more effectively educating patients and improving healthcare outcomes [4].

Most patients express significant preoperative anxiety, but it has been determined that the main source of this anxiety stems from concerns and fear regarding anesthesia and not surgery [5-9]. Patient anxiety has also been linked to healthcare outcomes, as preoperative anxiety has been shown to negatively impact anesthesia management and the postoperative period [4]. When it comes to what patients fear the most with anesthesia, many patients are afraid of postoperative pain, not waking up after surgery, postoperative fatigue, postoperative nausea and vomiting, and the anesthesiologist not attending to the patient [10-12].

To alleviate patient concerns, studies have shown that an improved doctor-patient relationship and time with the anesthesiologist are important [10,11,13,14]. Patients have also reported that they talked about their fears with someone, most often a relative, suggesting the need to improve the doctor-patient relationship [10,13].

Patient education literature is often written well above the recommended grade level, and thus many patients likely lack important information regarding GA [15,16]. There has been a push for anesthesiologists to spend more time educating their patients and utilizing more effective methods of education [11,12,14,17]. Examples of such methods include assessments of the effectiveness of various types of media (internet, videos, audiotapes, pamphlets, and booklets) [18,19]. There has been a large focus of research on video-assisted patient education, with results suggesting significant improvements [20,21]. A systematic review of media-based interventions found that patients who viewed education videos thought them helpful [18]. Much of the current literature on the impact and concerns of preoperative anxiety that patients have regarding GA appears to be outdated. We believe that the results of this survey will provide more up-to-date insight into patients' opinions, concerns, and beliefs.

## Materials and methods

In this descriptive study, we aimed to collect insight into patients' opinions, concerns, and beliefs when it comes to GA before and after administration via a 16-item Qualtrics survey. The exact survey questions and their order can be found in the appendix. Multiple physicians in Alabama and Florida were recruited to direct their patients to fill out the survey. These physicians were provided with recruitment flyers to post at their offices for patients to see or to directly provide patients with. Additionally, these flyers were posted in various healthcare facilities with permission in Alabama and Florida. The survey was also posted online via various social media platforms, including Reddit, Twitter, and Facebook. The survey was available for patients to fill out from May 2022 through September 2022. The survey was reviewed and approved by the Alabama College of Osteopathic Medicine Institutional Review Board (approval number: HS220404-E).

The survey included several initial screening questions to determine if the participant fulfilled the inclusion and exclusion criteria and to obtain informed consent. If it was determined that the participant did not fulfill the requirements, the survey would automatically close. The survey was completely anonymous and confidential. No identifying information was collected. The survey did ask questions regarding minimal personal health information and level of anxiety due to GA using the numerical rating scale. If a participant indicated that they had not undergone GA but would within the next six months, only their initial opinion of GA was collected. All statistical analysis of the collected data was performed in Microsoft Excel (Microsoft Corporation, WA, USA). All analyses of the variables on perceived levels of anxiety and danger were performed using a Student's t-test in Excel.

Subjects were included if they were 21 years old and older and if they had previous experience with GA or were undergoing GA within six months. Anyone who had not previously undergone a procedure using GA or who was not planning on undergoing such a procedure within the next six months was excluded.

The survey first asked for consent to undergo the questionnaire and then the age of the respondent. Respondents were then asked if they either had experience with GA or were going to undergo GA in the near future. If all of these questions were answered positively, they were then asked for further demographic information, such as self-reported birth sex, although participants were given the option to decline a response. Respondents were then asked if they had family or friends who had experienced GA and, if so, whether their opinion of GA had been affected by it. They were then asked about levels of perceived anxiety and danger before exposure to GA and if they had considered canceling the procedure because of that. Levels of perceived anxiety and danger were measured with a Likert scale from 0 to 10, where 0 was no perceived anxiety/danger and 10 was extreme perceived anxiety/danger. These questions were then repeated, but this time respondents were asked about their opinion after being exposed to GA, again with a scale from 0-10. Respondents were then asked how they would describe the experience of GA to family and friends. After this, they were asked whether they believed they had experienced any side effects and, if so, which ones they had experienced. In this question, a number of side effects were presented as options as well as a free response box. Finally, respondents were asked what interventions they believed would help them feel more comfortable with the experience of undergoing GA. In this question, a number of interventions were presented as options, as well as a free response box. The exact wording of the questions and the order in which they appeared are available in the Appendices section.

## Results

The survey received a total of 217 responses. After removing results that answered the entire survey in less than 80 seconds, had not completed the survey, or had not or would not undergo a procedure within six months, we were left with 157 (72%) responses. Only two people indicated they would not undergo a procedure within the next six months, and the rest were thanked for their time and not permitted to complete the survey based on our exclusion criteria.

The average age of respondents was 32.4 years old (range 21-68), with male respondents being 31.2% (n=49), female respondents being 61.1% (n=96), intersex respondents being <1% (n=1), non-binary respondents being <1% (n=1), and those who preferred not to disclose being 6.4% (n=10).

Subjects were asked if their thoughts and/or opinions of anesthesia had been influenced by others prior to undergoing GA. Of the 157 people, 16 answered this question. Of these, 10 said they had heard positive things and were comforted. Most of the positive feedback was from family members, who said they simply remembered induction and then had no memories until waking up refreshed after their surgery with no issues. Five people said they received negative feedback from other people or were made more anxious about the process. One person recounted being told of a family member having a serious allergic reaction post-induction and another account of someone waking up during a procedure. Two other respondents were told about negative post-anesthesia reactions, including talking excessively, disclosing too much personal information, becoming agitated, and assaulting a healthcare worker involuntarily. One final response was very nonspecific and could not be categorized as positive or negative.

The survey participants were asked about their level of anxiety and perception of danger before undergoing GA. The levels of anxiety and danger (on a scale of 1 to 10) before undergoing GA were 4.86 ± 2.76 (n=153) and 2.90 ± 2.50 (n=154), respectively (Table 1). Due to the perceived anxiety and danger of GA, 2.5% of respondents (n=4) considered canceling a future procedure due to concerns regarding GA.

**Table 1:** Self-reported level of perceived anxiety and danger before undergoing general anesthesia (GA) This information was self-reported by participants using a numerical scale (0-10), where 0 meant no anxiety at all, and 10 was extreme anxiety. Self-perceived levels of danger were measured using the same scale

Before experience	Level of anxiety	Level of perceived danger
0	7	18
1	12	36
2	23	33
3	16	22
4	12	13
5	8	4
6	23	8
7	22	12
8	16	2
9	10	2
10	4	4
Total responses	153	154
Average	4.86 ± 2.76	2.90 ± 2.50

The respondents' perceived levels of anxiety and danger both decreased after undergoing GA. The level of anxiety and danger (on a scale of 1 to 10) after undergoing GA was 3.03 ± 2.48 (n=149), a decrease of 1.83 (p=0.0001), and 2.21 ± 2.07 (n=151), a decrease of 0.69 (p=0.0001), respectively (Table 2). Only 3.2% (n=5) of respondents indicated they would consider canceling a future procedure due to GA.

**Table 2:** Self-reported level of perceived anxiety and danger after undergoing general anesthesia (GA) Respondents indicated on a scale of 0-10 their level of perceived anxiety and perceived danger after undergoing a procedure requiring GA (0 = none, 10 = extreme anxiety/extreme danger)

After experience	Level of anxiety	Level of perceived danger
0	29	30
1	21	45
2	21	19
3	21	28
4	16	8
5	10	4
6	13	9
7	13	6
8	3	1
9	2	0
10	0	1
Total responses	149	151
Average	3.03 ± 2.48	2.21 ± 2.07

**Figure 1:**
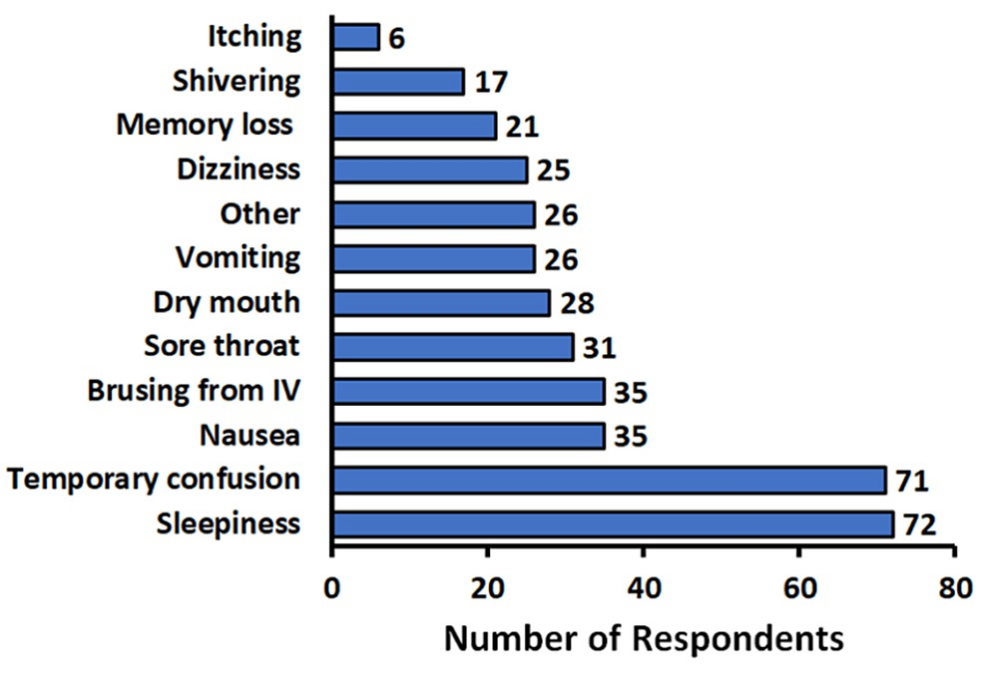
Patient self-reported effects from general anesthesia (GA) We asked participants, "Do you believe you experienced any side effects as a result of the anesthesia?" We provided a list of side effects participants could select from as well as an “other” option allowing for input from the participant

Survey participants were asked to self-report side effects from GA (Figure 1). A large majority of patients experienced minor side effects, with 86% (n=135) indicating said side effects. Sleepiness was reported by 45.9% (n=72) of respondents, temporary confusion was reported by 45.2% (n=71), nausea was reported by 22.3% (n=35), bruising from their IV lines was reported by 22.3% (n=35), and a sore throat was reported by 19.7% (n=31) of respondents. Among the “other” side effects reported, constipation was the only one cited more than once, with 1.9% (n=3) of respondents reporting that. Only a single person reported a very significant issue following anesthesia, which was temporary blindness that resolved after a few days.

**Figure 2:**
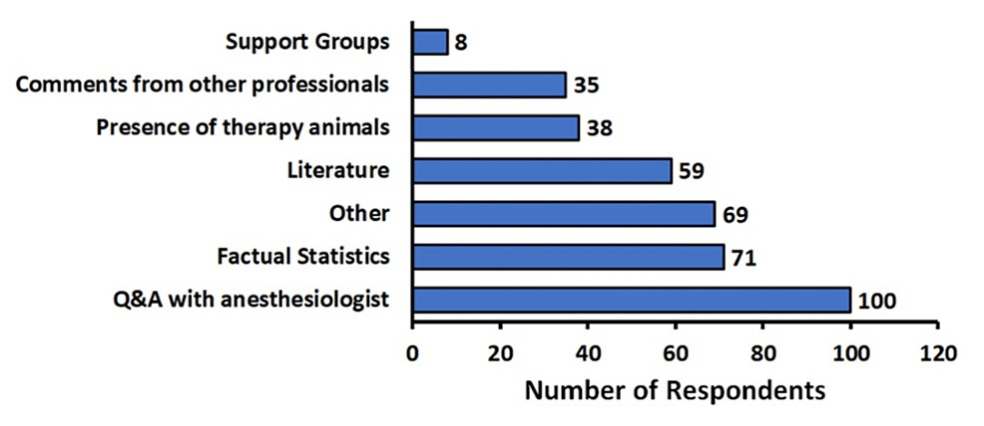
Perceived interventions that would improve participant level of comfort toward general anesthesia (GA) We asked participants, “What interventions do you believe would most likely help you feel more comfortable undergoing general anesthesia?” We provided a list of interventions participants could select from as well as an “other” option allowing for input from the participant

Respondents were asked what they believed would improve their comfort level with GA (Figure 2). The most frequent request was a questions and answers session with the anesthesiologist, with 62.7% (n=100) of respondents selecting this option, with statistics at 45.2% (n=71) and literature on the procedure at 37.6% (n=59) being the next two most frequent selections by respondents.

## Discussion

As any operation is certain to bring some risks, it is only logical that many people who are undergoing a procedure would have some concerns, and this assertion is backed up by previous research [5-9]. Our results showed that, while people initially had slight anxiety about undergoing GA (Table 1), most participants did not consider that anxiety significant enough to change their plans. The participant's level of anxiety prior to GA was higher than their level of perceived danger (Table 1). We interpret this as meaning that although patients were anxious about GA, most of them had some knowledge about it being generally safe and well tolerated.

Overall, very few people were anxious enough or perceived sufficient danger to consider canceling their procedure due to GA. This may be due to the existing interventions that anesthesiologists, surgeons, and hospitals take to provide support and reassurance. Our survey participants were also mostly younger, with an average age of 32.4 years old. This is likely due to the predominant method that was used to solicit responses to our survey, which was the social media platform Reddit. However, all recruitment methods required that our respondents had internet access and therefore may have had better information than the average person. Another major confounding factor here is the necessity or type of surgical procedure the subject was receiving, which is not something we inquired about and could potentially alter our results. We believe further inquiry in this field is warranted.

After undergoing GA, the average level of anxiety decreased (Tables 1-2). This was supported by the observation that most people also reported no significant side effects related to GA. It is, therefore, likely that much of the anxiety from before undergoing the procedure the first time is a fear of the unknown. Once patients have an experience with GA to draw upon, much of the anxiety is gone. As previous research has shown that preoperative anxiety has a negative effect on both the anesthesia procedure and the postoperative recovery period, it stands to reason that prior exposure to GA means that the procedure is likely to be better tolerated in future exposures [4].

Several people also elaborated that they spoke to acquaintances who had undergone GA, and in the majority of cases, those other people reassured the respondent. However, there were some cases in which shared opinions and experiences caused an increase in the level of concern of the respondent, usually when sharing some sort of negative reaction to GA. It is reasonable to assume that these patients would have a higher level of anxiety about GA before undergoing GA as compared to the general public, which, as previously noted, could potentially mean worse healthcare outcomes for these individuals [4].

We gathered responses as to what the best approach would be to reassure potential patients. The most chosen request was a discussion with the anesthesiologist. This result is consistent with previous explorations of the topic within the literature, and we believe our results bolster this burgeoning scientific consensus [10,11,13]. Therefore, facilitating and enhancing discussions with anesthesiologists could be explored by the medical community as a modality to mitigate anxiety before major procedures, especially since many patients often do not completely understand what GA entails [15]. The next best things to reduce anxiety were factual statistics about GA as well as literature (Figure 2). It may, therefore, be useful that prior to any surgical procedure, this kind of literature be passed on to patients while obtaining informed consent. One inexpensive and very simple intervention that was suggested by several survey respondents was to consider providing animal therapy pre-operatively.

The most common side effects of GA that were reported were temporary confusion and sleepiness. Other common side effects like nausea, dizziness, and irritation around the IV site were prominent but less likely overall (Figure 1). These common side effects resolved on their own and caused no significant distress or permanent impairment, which could be a useful thing to emphasize to patients. There was also one significant reaction reported, which was temporary blindness, but it is likely that this patient had underlying conditions that may have predisposed him or her to this issue.

The limitations of our study include that the sample size was smaller and did not perfectly reflect the general population that undergoes GA, as participants required internet access to complete the survey. Furthermore, many of our responses were collected through the website Reddit. While the user base there is broad, it is generally younger than the general population, which could skew the data. Additionally, we collected data regarding perceived side effects. These data were collected at a single point in time, so participants' recollections of perceptions prior to GA may not accurately reflect their true experiences. Finally, we did not differentiate between the types of surgery that were performed, as emergency major surgery is very different from an elective procedure. This could therefore skew opinions of the GA that is needed as well as the risk/reward ratio for GA. We believe that there is still substantial room for further investigation on this topic.

## Conclusions

Considering the frequency and importance of the use of GA, developing a better understanding of patient opinions will facilitate future advancements in the care of a significant proportion of the general public. We hope that our findings will encourage future research into this specific area and encourage healthcare professionals to think about patient perspectives that may be similar to some of the themes we have observed. For example, we had a significant number of respondents share that an anesthesiologist answering their questions would increase their level of comfort and is something that could easily be performed. Overall, our results clearly show that people generally have positive opinions about GA. However, we believe there is room for improvement in terms of patient education, comfort level, and safety. When it comes to healthcare outcomes and patient safety, even a small improvement is significant and something that all healthcare professionals should strive for.

## Appendices

1. “Do you consent to undergoing this survey?”

2. “What is your current age?”

a. This option contained a text box.

3. "Have you already undergone a procedure requiring general anesthesia? (If you have not, you will only be asked questions about your current opinions and not how they were modified)."

4. “Will you undergo a procedure requiring general anesthesia within the next 6 months?”

5. “What is your birth sex?”

a. This option contained a text box for self-disclosure as well as a prefer not to respond option.

6. “Has anyone in your surroundings (friends, family, etc.) that has undergone a procedure requiring anesthesia influenced your thoughts and/or opinions of anesthesia? If so, please explain. If not, please leave blank.”

a. This question contained a text box for free response.

7. “What was your level of perceived danger to yourself before the procedure? 0 = none. 10 = extreme danger. - perceived danger”

8. “Did you consider canceling or not undergoing the procedure because of general anesthesia?”

9. “What is your level of perceived anxiety after the procedure if you would have to undergo it again. 0 = none. 10 = extreme anxiety. - Perceived anxiety”

10. “What is your level of perceived danger to yourself after the procedure if you would have to undergo it again. 0 = none. 10 = extreme danger. - perceived danger”

11. “Would you consider canceling or not undergoing a further procedure because of general anesthesia?”

12. “How would you describe your experience with anesthesia to friends and family?”

a. This question contained a text box for free response.

13. Do you believe you experienced any side effects as a result of the anesthesia? Select all that apply - Selected Choice

a. This question had the following options as well as a free text response box:

i. Nausea

ii. Vomiting

iii. Sore throat

iv. Bruising or soreness from an IV drip

v. Dizziness

vi. Temporary confusion

vii. Sleepiness

viii. Difficulty passing urine

ix. Dry mouth

x. Shivering

xi. Muscle aches

14. “What interventions do you believe would most likely help you feel more comfortable undergoing general anesthesia? - Selected choice”

a. This question had the following options as well as a free text response box:

i. Presence of therapy animals in the office

ii. Q&A session with anesthesiologist

iii. Being given literature on the topic (flyers, pamphlets)

iv. Thoughts and comments from other professionals

v. Factual statistics showing risks of adverse effects

vi. Support groups with other people who have undergone general anesthesia
